# Security of ADS-B and Remote ID Systems: Cyberattacks, Detection Techniques, and Countermeasures

**DOI:** 10.3390/s26020634

**Published:** 2026-01-17

**Authors:** Qinxuan Shi, Toro Dama Caleb, Sicong Shao, Naima Kaabouch

**Affiliations:** School of Electrical Engineering and Computer Science, University of North Dakota, Grand Forks, ND 58202, USA; qinxuan.shi@und.edu (Q.S.); toro.caleb@und.edu (T.D.C.)

**Keywords:** ADS-B, Remote ID, unmanned aerial system, cybersecurity, risk analysis, attack detection, attack countermeasures

## Abstract

The aviation sector relies on cooperative surveillance systems such as Automatic Dependent Surveillance-Broadcast (ADS-B) and Remote Identification (RID) to enhance safety and efficiency. However, their open, unencrypted communication protocols make them vulnerable to various cyberattacks. This survey examines the current state of knowledge on attacks, detection techniques, and countermeasures for both ADS-B and RID, addressing a gap in the literature by analyzing them side by side. It categorizes attacks, including emerging threats, reviews detection methods from traditional to modern AI-based approaches and discusses existing countermeasures. Furthermore, this paper provides a list of simulation tools and open-access datasets and identifies current research challenges and future directions.

## 1. Introduction

The aviation sector has experienced continued growth in recent decades, driven by its impact on the global economy and other critical sectors. According to the International Civil Aviation Organization (ICAO), a United Nations agency that supports 193 countries to cooperate and share their skies for mutual benefit, 200,000 flights per day are estimated by mid-2030 [1]. In addition, the Federal Aviation Administration (FAA) forecasts that the commercial fleet for Unmanned Aircraft Systems (UAS) will reach 858,000 units by 2026 and recreational small Unmanned Aerial Vehicles (UAVs) to reach 1.81 million units [2]. To secure the NAS, the global airspace relies fundamentally on cooperative surveillance systems to maintain safety, efficiency, and accountability. At the core of this infrastructure are two critical devices, yet inherently vulnerable: the Automatic Dependent Surveillance-Broadcast (ADS-B) system, which serves as the backbone for Air Traffic Management (ATM), and Remote Identification (RID) which manages UAV operations within the UAS Traffic Management (UTM) ecosystem A study investigated the impact of ADS-B on general aviation and found a 53% reduction in the likelihood of an accident and a 89% reduction in the possibility of a fatal accident for aircraft using ADS-B in [3].

ADS-B relies on aircraft autonomously broadcasting their precise position via Global Navigation Satellite System (GNSS) inputs, along with velocity data and identity over open, unencrypted data links. This unencrypted architecture, driven by a historical priority on simplicity and safety over security, results in ADS-B being susceptible to numerous cyber threats [4,5]. Similarly, RID emerged as an identification requirement for the safe integration of small UAVs into the national airspace, particularly for Beyond Visual Line-of-Sight (BVLOS) operations [6]. RID establishes an electronic identification process requiring UAV operators to register and continuously broadcast their drone’s key data, including the unique UAV ID, location, speed, and timestamp, for traceability and monitoring. Like ADS-B, RID transmits plain-text messages, which creates an inherent vulnerability to the same types of cyberattacks [7].

The shared vulnerability of these systems stems from their reliance on open, unencrypted wireless communication, making them susceptible to cyberattacks. Despite the importance of secure cooperative surveillance, most survey papers focused on ADS-B and very few on RID. For instance, a recent survey provided a deep analysis of ADS-B security or Artificial Intelligence (AI)-based detection for ADS-B [8], but omitted a parallel analysis of the RID security. Other works that compare the systems often focus on traffic management interoperability rather than a holistic cybersecurity framework.

This survey aims to address this gap by consolidating the current state of knowledge regarding attacks, detection techniques, and countermeasures for ADS-B and RID. Such an integrated approach is necessary, given that both systems share a fundamental vulnerability and face increasing threats, including the rise of Adversarial Machine Learning (AML) attacks in wireless communications. In addition, reviewing both ADS-B and RID side-by-side allows for a comprehensive understanding of the entire air traffic threat landscape, especially as the attacker base has risen from the ground to the air due to the rapid growth of UAV traffic [9].

This paper provides a comprehensive survey of the security risks facing ADS-B and RID, detailing the spectrum of attacks that exploit their open architecture. We review various attack types targeting ADS-B and RID. Furthermore, we analyzed existing detection mechanisms and countermeasures, focusing on both traditional verification methodologies and AI/machine learning (ML)-based methods designed to safeguard the integrity, confidentiality, and availability of important air traffic data in both manned and unmanned airspace, and provided some insights into future directions.

The key contributions of this paper are as follows:

1. It fills a gap in the literature by providing the first consolidated survey that comprehensively analyzes the security landscapes of both ADS-B and RID systems side by side.
2. It presents a detailed categorization of cybersecurity threats applicable to both protocols, including an in-depth analysis of emerging attack vectors such as AML against AI-based detection systems, and a detailed analysis of RID-specific spoofing attacks that leverage low-cost, off-the-shelf hardware.
3. It delivers a thorough review of modern attack detection techniques, comparing non-ML, traditional ML, and advanced deep learning (DL) methods.
4. It analyzes countermeasures, including practical and protocol-agnostic methods, such as fingerprinting, multilateration, and data fusion, that can enhance the security of both systems without requiring fundamental changes to their existing architecture.
5. It provides a valuable resource for the research community by compiling and discussing available simulation tools and public datasets for developing and validating new security solutions for ADS-B and RID, and analyzing the specific gaps in available RID attack datasets for validating new security solutions.

The remainder of the paper is structured as follows. Section 2 presents the underlying concepts of ADS-B and RID. Section 3 summarizes previous surveys and reviews related to ADS-B and RID cybersecurity. Section 4 discusses cybersecurity attacks targeting both systems, highlighting how each system is impacted. Section 5 provides a survey of attack detection methods. Section 6 discusses countermeasures to mitigate cyberattacks. Section 7 discusses relevant tools and datasets available to researchers. Finally, Section 8 discussed some of the challenges and future directions.

## 2. Fundamentals of ADS-B and RID

The purpose of ADS-B is to provide continuous positional and navigational awareness for both ground controllers and pilots [5,10]. RID, conversely, emerged as a critical need for integrating small UAVs into the national airspace [11]. RID operates within the UTM framework, focusing on accountability and traceability for UAVs, particularly in the very low-level airspace. Both ADS-B and RID rely on aircraft determining and broadcasting their current status, including identification and location, establishing foundational elements for traffic monitoring and ensuring safety across respective domains [9,12]. ADS-B evolved as an extension of the existing Mode-S Secondary Surveillance Radar (SSR) framework [4,13], whereas RID establishes new communication links tailored to resource-constrained UAV networks, enabling RID through both direct broadcast and network connectivity [14,15]. This section provides the fundamental concepts of ADS-B and RID.

### 2.1. ADS-B

**Figure 1:**
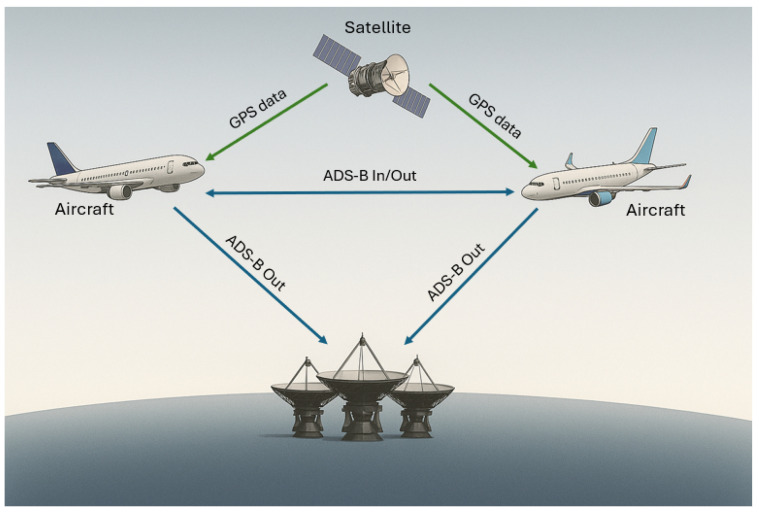
Overview of the ADS-B system.

The operational principle of ADS-B is defined by two key terms: “automatic” and “dependent”. It is dependent because the aircraft relies on GNSS inputs, such as Global Positioning System (GPS), to autonomously and accurately determine its position, combining it with velocity, altitude, and identity information gathered from other onboard systems. It is automatic because the aircraft’s ADS-B Out transmitter continuously broadcasts this synthesized flight data without requiring intervention from the pilot or air traffic controller. As shown in Figure 1, an aircraft uses GPS-derived position and status information to continuously broadcast ADS-B messages containing key fields such as identity, altitude, speed, and climb/descent rate. These messages are received by nearby aircraft and ground stations via ADS-B In receivers to support traffic monitoring and situational awareness. ADS-B is designed to enable cooperation, situational awareness, and safety among aircraft, thereby enhancing air traffic management without requiring pilot or controller intervention. It also provides improved accuracy compared to radar, and its accuracy does not deteriorate as the range from the receiver expands [16].

**Figure 2:**
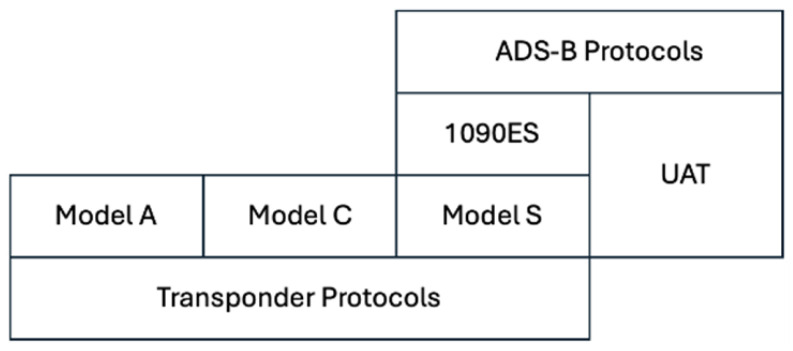
Specification hierarchy of ADS-B.

Historically, air traffic surveillance relied on Primary Surveillance Radar, a non-cooperative technique that detected objects via EM wave reflection [13,17] and SSR, a partly cooperative technique relying on transponders installed on aircraft [4,5]. SSR operates on an interactive “interrogation-response” model, where ground stations transmit coded interrogation signals to aircraft [17]. As shown in Figure 2, earlier SSR utilized Mode-A, a 4-octal-digit squawk code, and Mode-C, which provides barometric altitude reporting [4,9,13]. Due to increasing air traffic density, these modes experienced limitations such as signal overlap, also known as synchronous garbling, and False Replies Unsynchronized in Time [9]. To mitigate these issues, the Mode Select Beacon System (Mode-S) was developed, functioning on selective interrogations to gather varied information from specific aircraft. Mode-S operates using 1030 MHz for ground-to-air interrogations and 1090 MHz for air-to-ground replies [17]. Mode-S transponders incorporate 256 data registers updated by the aircraft’s systems. As illustrated in Figure 2, ADS-B is supported by two data links to enhance interoperability, regulatory compliance, and legacy support. First is the 1090 MHz Mode-S Extended Squitter (1090ES), which is a technological upgrade to SSR and an extension of the Mode-S protocol, implemented directly into existing Mode-S transponders via the “extended squitter” feature [9,13,18,19]. Second is the 978 MHz Universal Access Transceiver (UAT), which was specifically designed for ADS-B and complementary aviation services, such as Traffic Information Service-Broadcast and Flight Information Service-Broadcast [17]. However, this will require installing new hardware, unlike the 1090ES, which is widely used because it is implemented in existing Mode-S transponders already installed in aircraft. Another distinct feature between the two ADS-B data links is reflected in message size: the UAT message data block is 272 bits, whereas the 1090ES is 112 bits [18]. This explains why UAT can accommodate complementary aviation services information as discussed earlier.

The coexistence of these two links is critical; hence, the system design includes a supplementary component called Automatic Dependent Surveillance-Rebroadcast (ADS-R). ADS-R is deployed in conjunction with ADS-B to receive traffic information broadcasts on one link (either 1090 MHz or 978 MHz) and rebroadcast the information to users operating on the opposite data link frequency, thereby facilitating interoperability [4,12].

**Figure 3:**
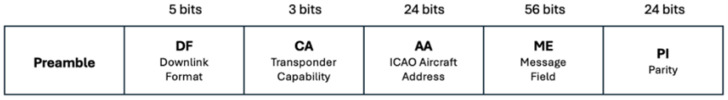
ADS-B data format.

The ADS-B frame format for the widely used 1090ES datalink is shown in Figure 3. It uses a standard message format, comprising a preamble followed by a 112-bit message, including downlink format (DF), transponder capability (CA), ICAO aircraft address (AA), ADS-B message field (ME), and parity check (PI), as illustrated in Table 1. The DF field specifies the message type. When DF is set to 17, the message is classified as an extended squitter, allowing for the transmission of 56 arbitrary bits in the ME field. The CA field describes the operational capabilities of the Mode S transponder. The AA field contains a unique 24-bit ICAO aircraft address used for aircraft tracking and identification. The ME field carries the ADS-B data payload, while the PI field provides a 24-bit parity code that supports error detection and correction. By using the parity information field with a fixed generator polynomial of degree 24, ADS-B receivers are able to correct up to 5-bit errors within 1090ES messages, enhancing the reliability and integrity of the transmitted data [4,18,20,21].

### 2.2. Remote Identification

**Figure 4:**
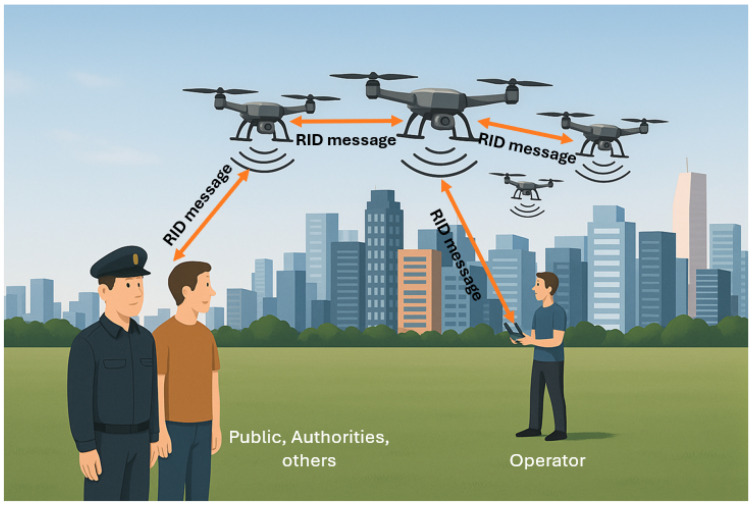
Illustration of RID for UAVs.

RID is an important and evolving technology essential for the safe and efficient integration of UAVs into the low-altitude airspace, particularly for operations like Urban Air Mobility and BVLOS flights [22,23]. RID functions as a digital license plate for UAV intended for identification and compliance [24]. This enables regulatory authorities (e.g., the FAA), operators, and other airspace users to identify and track UAVs in real time, improving accountability and airspace safety. As illustrated in Figure 4, RID devices broadcast standardized identification and location data that authorized parties can receive for monitoring and compliance. Due to the inherent safety and security concerns surrounding UAV operations, RID has reached technical, legislative, and regulatory maturity in jurisdictions like the US, established by the FAA and the EU, managed by the European Union Aviation Safety Agency [23]. The implementation of RID is extensively standardized, most notably by the ASTM F3411-22a [22], which covers the performance requirements for RID of UAV operating in the Very Low Level airspace over diverse environments. The ASTM standard defines the overall conceptual framework, messaging formats (known as “Open Drone ID”), transport mechanisms, and minimum performance standards for both broadcast-based and network-based RID [11].

While this ASTM [22] standard defines the data transmission protocols, a parallel and critical framework for authentication is being developed by the Internet Engineering Task Force Drone RID Protocol Working Group [25]. This work aims to leverage existing Internet resources to support RID functions beyond mere identification, such as enabling contact between authorized parties and the remote pilot, providing strong authentication, and expanding support for applications like air traffic control.

These RID devices are either integrated into the UAV or externally attached. They broadcast unique identification messages as mandated by the FAA’s RID rule [26]. The prerequisite for utilizing RID is the registration of the UAV node and/or its operator with the regulatory authority, such as the FAA. Once registered, the authority assigns a unique ID to the UAV [27]. The core function of RID is the continuous dissemination of essential flight data. The broadcast consists of both static and dynamic data. As shown in Table 2, static messages containing unchangeable data, such as the UAV’s unique identification number or session ID, transmitted every three seconds while dynamic data consisting of the UAV’s current position, altitude, and velocity, which changes during the duration of the flight, are transmitted at least every one second [11,28]. However, RID relies on GPS for its position data; therefore, its accuracy can be affected by incorrect GPS data in GPS spoofing scenarios.

The communication modalities used in deploying RID can be broadcast-based or network-based [11,24]. Broadcast RID (BRID) relies on a unidirectional, one-way local data link transmission where the UAV continuously transmits RID data using radio signals directly to receivers in the vicinity [22,25]. The data broadcast has no specific destination or recipient and can be received by anyone within the broadcast range using commonly carried handheld devices such as smartphones and tablets. BRID is crucial in areas with unreliable, disrupted, or unavailable network coverage. As shown in Table 3, RID depends on wireless communication mediums such as Bluetooth, Wi-Fi, or Low Power Wide Area Network (LoRa). The Bluetooth 4.x payload is limited to 25 bytes for Open Drone ID messages; the full complement of broadcast data cannot fit in a single message, requiring multiple small messages or pages. These messages must be correlated, potentially using the Media Access Control (MAC) address, although MAC addresses can be randomized or spoofed, for data links that can encapsulate multiple messages in a single frame such as Bluetooth 5.x and Wi-Fi, a Message Pack (Message Type 0xF) is used, containing concatenated messages [22,25]. Additionally, the effectiveness of BRID depends on the range of the broadcast medium used, as illustrated in Table 3.

Network RID (NRID), on the other hand, is used when both UAV operations and end-users of RID display applications have access to the Internet, typically via a cellular network. Unlike BRID, NRID allows for UAV identification information to be made available globally and indirectly via servers. The only range limitation is the UAV’s access to the network; as long as the UAV and the intended receiver can reach the cellular network, the range has no restriction. The FAA has adopted BRID rather than NRID for security reasons [29]. A significant challenge for RID, as with the ADS-B system, is that it is fundamentally vulnerable because its broadcast messages are transmitted in clear text without inherent cryptographic protection for message integrity and authenticity [11,12]. Table 4 provides a technical comparison for ADS-B and RID.

## 3. Related Work

Numerous existing survey papers review the security of ADS-B, covering challenges arising from its unencrypted, unauthenticated wireless transmission between aircraft and ground stations, potential solutions for attack detection or defense mechanisms, and future research directions. However, there are fewer dedicated to the security of RID. Table 5 lists recent surveys on ADS-B and RID cybersecurity and highlights the topics covered fully, partially, or not at all. These surveys were analyzed if they covered several security related topics, such as cyber attacks, detection techniques and countermeasures.

For instance, the authors of [18] focused on analyzing ADS-B vulnerabilities in the ADS-B protocol stack and ADS-B security requirements. In addition, they conducted a comparative analysis of existing countermeasure techniques, which revealed the inefficiency and infeasibility of these methods. Similarly, a review by [30] examined different ADS-B attacks on ADS-B, their possibilities of implementation and associated impact. They also summarized some countermeasure techniques and stated that most of these techniques are not feasible due to the lack of backward compatibility, the high cost of implementation, and the introduction of new threats.

Building on the understanding that ADS-B can be attacked, some studies have focused on analyzing specific methodologies for attack detection, particularly through data driven approaches. For instance [32] investigated the challenges associated with obtaining real world attack data by examining threat models and construction strategies capable of generating high fidelity attack datasets, which are essential for training detection algorithms. Ref. [12] focused on attack detection, proposing techniques to detect these attacks. First, they reviewed common attack models and detection techniques; then they proposed a structured, multilayered detection strategy involving both aircraft and ground stations, leveraging sequential and collaborative techniques with the focus of improving the positive detection ratio. In addition, ref. [33] reviewed the application of machine learning techniques for attack classification, demonstrating the effectiveness of multi class classifiers. Their work showed that machine learning can be effective in automatically identifying the nature of an attack, thereby providing more granular situational awareness than simple anomaly detection.

In [31], the authors classified ADS-B vulnerabilities into two vulnerability based categories: attack intention and security requirements. Vulnerability based on attack intention considers the attacker’s primary motivation beyond the ADS-B attack itself. The vulnerability based on security requirements focus on integrity, confidentiality, availability and authentication of ADS-B data. In this paper, the authors recommended solutions based on blockchain technology to achieve secure authentication and protection of ADS-B messages, along with deep learning-based anomaly detection systems. Ref. [35] presented a comprehensive review of aircraft systems and their networks, with a focus on the cyber threats they are exposed to and associated impact. Furthermore, they provided a structured taxonomy that extends the MITRE ATT&CK framework specifically for avionics.

Ref. [36] reviewed recent developments in ADS-B implementation after the mandate of 2020 by considering relevant documentation from regulatory authorities such as ICAO, FAA, and Eurocontrol, in addition to existing research. They also reviewed blockchain techniques as one of the countermeasure techniques for location verification in addition to techniques such as multi-lateration surveillance, Kalman filtering, and data fusion. In [37], the author evaluated the performance of various defense mechanisms proposed in the literature, highlighting trade-offs among latency, scalability, and cost. Furthermore, the author discussed the barriers to these approaches in real-world implementation.

The authors of [34] provided an overview of the ADS-B system to assist with a comprehensive vulnerability assessment. They also reviewed the current state of ADS-B implementation, future directions, and associated challenges. Additionally, the authors of [38] discussed existing vulnerabilities in ADS-B for a better understanding of associated threats. They also surveyed potential mitigation solutions such as encryption and authentication. In [39], the authors reviewed existing research related to the techniques used as protective measures to preserve the integrity and trustworthiness of ADS data broadcasted by aircraft to ground stations and other aircrafts around them. They evaluated these techniques with the aim of identifying gaps for further research to strengthen the data integrity of ADS-B systems against threats posed by spoofed traffic ushered into the ADS-B network.

Regarding RID, very few survey papers have been published. For instance, the authors of [11] surveyed the current landscape of RID by reviewing the regulatory and standardization efforts across various countries and organizations. In addition, they highlighted the underlying technologies for RID, such as Wi-Fi, Bluetooth, and cellular networks, and discussed some existing industrial solutions. In [20], the authors provided an integrated review of both ADS-B and RID showing their similarities and differences within the UTM. They examined the definitions, data formats, and technologies for the legacy ADS-B used in manned aviation to newer RID standards based on Bluetooth or Wi-Fi. They conducted a structured comparison of the strengths and weaknesses of these systems, identifying challenges in security, safety, and communication for each. They also proposed a multi-channel communication architecture as a solution to enhance reliability and availability.

The integrated security challenges of both ADS-B and RID as communication systems related to surveillance and identification within UTM are often addressed partially in surveys focused on UTM security. For instance, the authors of [14] discussed the security requirements for communication links involving ADS-B and RID to enable safe traffic management. Similarly, ref. [15] details UTM services, including ADS-B and RID communication protocols, noting their inherent security challenges and limitations.

Following a comprehensive review of existing surveys, much of the focus has been on investigating ADS-B vulnerabilities and countermeasures. Although some UTM cybersecurity surveys combine ADS-B and RID to show their unique relationship and evolving coexistence within the UTM ecosystem, there is a need for a focused, comprehensive survey that fully covers every aspect of their security provided a comprehensive, side-by-side analysis of working principles, attack techniques, detection, and countermeasures of both ADS-B and RID. This paper aims to fill this gap by providing an updated literature review of the working principles, attack types, detection techniques, and countermeasures for attacks targeting both ADS-B and RID. In addition, this paper presents a review of available tools and datasets, identifies gaps in current research, and provides some actionable directions for future work.

## 4. Cybersecurity Threats

Due to their inherent reliance on wireless communications, ADS-B and RID devices are prone to various types of cyberattacks. While both ADS-B and RID share the vulnerability of unencrypted broadcast, their divergent physical and link layers necessitate fundamentally distinct attack methodologies. ADS-B operates on the 1090 MHz frequency using pulse position modulation, where data is encoded strictly via the timing and presence of RF pulses. This analog reliance exposes ADS-B to unique physical layer signal manipulation techniques such as overshadowing and bit-flipping, where an adversary transmits a high power signal to replace legitimate messages or superimpose fake signals to alter binary values during transmission [5,18,19].

In contrast, RID devices utilize consumer grade wireless standards such as Wi-Fi and Bluetooth operating in the 2.4 GHz or 5.8 GHz frequency bands, shifting the attack surface from RF pulse manipulation to digital link layer exploits [11,22]. Unlike the stateless broadcast of ADS-B, RID implementations using Wi-Fi are susceptible to protocol specific attacks inherited from standard computing networks, such as de-authentication, where spoofed management frames force a disconnection between the UAV and its controller, a vector nonexistent in the connectionless ADS-B protocol [14,24]. Furthermore, while ADS-B injection requires modulating RF signals to mimic Mode-S squitters, RID injection exploits the IEEE 802.11 or Bluetooth frame structures, allowing attackers to inject malicious payloads directly into vendor specific elements within beacon management frames without needing to overpower physical signals.

The attacks can be classified into several categories: Denial-of-Service/Distributed Denial-of-Service (DoS/DDoS), Spoofing, Injection, Intrusion, Adversary-in-the-Middle (AitM), Supply Chain, and Adversarial attacks. A brief description of each of these categories is given below. Table 6 summarizes the attack categories, their descriptions, and their impacts.

DoS/DDoS seek to make communication resources unavailable or degrade system performance to the point of operational failure. The primary technique for implementing a DoS/DDoS attack on RID and ADS-B is jamming [11,40,41,42]. The intent of jamming RID is to disrupt either the transmission or reception of RID messages. A successful DoS/DDoS attack can effectively render a UAV invisible to authorized entities, preventing its identification and tracking. A broader network interruption as the case of DDoS could cause disruption to the UTM services, potentially leading to mission failures and a loss of airspace control [24,43]. Similarly, ADS-B jamming involves the deliberate transmission of signals [44,45,46] to hinder the reception or transmission of legitimate ADS-B data. Operating on the 1090 MHz frequency, this attack can also jam a ground contol station, which blocks the reception of all ADS-B signals intended for that ground control station, with localized impact determined by the jamming signal range and proximity [16,18,46], or aircraft flood denial that targets individual aircraft, which is challenging to execute from the ground unless positioned near airports during critical operations like takeoff and landing [4,18].

Spoofing involves an attacker presenting a false identity or modifying original content with the intent of misleading systems and operators via fraudulent RID, ADS-B, or GPS signals. RID spoofing broadcasts falsified identification packets intended to impersonate legitimate UAVs, compromising recognition and tracking mechanisms and leading to false classifications and misidentifications among observers and neighboring UAVs [11,47]. ADS-B spoofing shares the same similarities with RID spoofing in terms of the objectives and impact since it also involves falsifying ADS-B messages that attempt to imitate authentic messages, which leads to counterfeit broadcast of ADS-B data [48,49,50]. Unlike jamming, spoofing injects hazardously misleading information, creating significant errors in an aircraft’s reported position, velocity, and time data without the receiver detecting the information as untrustworthy [49,51]. The impact can range from altering the reported trajectories of real aircraft to causing operational disruptions, mission failures and risk of collisions. While, GPS spoofing involves an attacker transmitting fake, higher-power GPS signals [52,53,54], causing the receiver to calculate an incorrect position that is then broadcast via both RID or ADS-B [55,56,57], indirectly modifying their outgoing messages and potentially resulting in path deviation, mission failure, or collisions.

RID injection transmits fake RID frames containing valid attributes such as velocity, location, and identification number, making it difficult for stakeholders, including regulatory agencies and Ground Control Station (GCS) operators, to distinguish counterfeit messages from original ones, thereby disrupting the core function of accurate UAV identification [26,58]. Similarly, in ADS-B injection, the attacker broadcasts modified or fake ADS-B messages [4,18,59]. The impact is detrimental because fake and modified messages are often perceived as legitimate by recipients, leading to operational disruptions and an increased risk of collisions. Another form of injection attacks is the ghost injection attack, which creates fake aircraft by broadcasting entirely new fake ADS-B and RID messages [59,60]. These fake aircraft can confuse operators, overwhelm UTM systems with false data, and force pilots to alter flight paths, ultimately compromising aviation safety [15,61].

An Intrusion attack involves gaining unauthorized access to the underlying hardware, firmware, or software stack of the RID or ADS-B system. These categories include malware infection, firmware flashing/modification, and fuzzing [41,62,63,64,65]. Malware infection involves the insertion of malicious code into onboard systems of the UAV with the objective of exploiting software vulnerabilities to alter program execution or gain unauthorized access. This, in turn, impacts the data integrity of both ADS-B and RID, causing system overload through denial of service, component failure, or even complete destruction of the UAV [41,62,63,66,67]. Firmware modification involves unauthorized alteration of the embedded software in a system’s firmware which controls its operational behavior [68,69,70,71]. In RID, this could alter the identifiers being broadcast, cloak the UAV from detection, or substitute randomized identities. In ADS-B, intrusion can lead to false positional data, suppress valid outputs, or replay cached messages, leading to the generation of ghost aircraft and degrading surveillance data reliability. Fuzzing attacks involve sending large volumes of malformed, unexpected, or random data to ADS-B and RID receivers, as well as to GCS. The goal is to reveal weaknesses such as crashes, misbehavior, or failure to validate broadcast messages without providing specific exploitation techniques.

An AitM attack occurs when a malicious actor intercepts and alters messages exchanged between two or more systems, compromising data integrity and leading to unauthorized control of airspace. AitM often takes the form of replay attacks, Man-in-the-Middle (MitM), and session hijacking. Replay attack for both ADS-B and RID shares similarities. The attacker uses a low-cost Software Defined Radio unit to passively monitor the specific RF spectrum constituting the Wi-Fi/Bluetooth frequencies for RID [20], or the 1090/978 MHz frequency for ADS-B frequency [49,72], and record these authentic messages then re-sent them into the airspace [73,74,75]. These hey messages are difficult to identify, which can lead to degraded situational awareness and disruption of airspace safety. The Man-in-the-Middle attacks involve a third party actively intercepting and gaining control of the flow of data within the communication channel [24,76,77,78,79,80], allowing the attacker to intercept, modify, change, or replace communication traffic within the RF spectrum for either RID or ADS-B, thereby compromising message integrity. The overall impact threatens the safety and reliability of the air traffic network, as false information could lead to mid-air collisions.

Supply Chain Attacks exploit hardware and software vulnerabilities introduced by third-party vendors involved in the design and distribution of RID and ADS-B modules and their embedded firmwares. These are stealthy physical attacks embedded before components are deployed [81,82]. Types of supply chain attacks include hardware supply chain and software supply chain. In Hardware supply chain attack, the malicious modifications are introduced to hardware components at any stage of the supply chain, from design to manufacturing and distribution [83]. This is achieved through the intentional introduction of malicious modifications or “Trojans” into integrated circuits. For the RID module, this compromise could allow an attacker to inject false information directly into the UAV’s flight controller, potentially resulting in a complete takeover. For an ADS-B transponder, a malicious hardware component could inject false data into the outgoing signal or modify received data before processing, compromising data integrity at the source [31,37]. A Software supply chain attack involves injecting malicious code into the software product during its lifecycle including design, development, or updates. An adversary can infiltrate a software developer’s network to introduce malicious code into a software update for RID and ADS-B receivers or transponders, which once deployed, serves as a vector to execute a variety of subsequent attacks.

AML attacks target AI models used to process and interpret RID and ADS-B data for advanced functionalities and anomaly detection [84,85,86]. Types of AML attacks include Poisoning, Evasion, Model Stealing, Model Inversion, and Membership Inference. Poisoning attacks involve injecting corrupted data into the ML training dataset [87,88]. Poisoning attacks on ADS-B or RID based intrusion detection systems (IDS) can be achieved by feeding corrupted traffic into data streams or logs used for model training, degrading detection performance and leading to unsafe operational decisions. In Evasion attacks, the attacker subtly manipulates the input test data [89,90]. In the context of ADS-B and RID, an evasion attack would involve crafting a subtly perturbed ADS-B or RID message which, despite carrying malicious information such as spoofed position data or a ghost aircraft message, is designed to be classified as legitimate by the target AI model. When fed to AI based detection mechanisms in ADS-B and RID, they allow for other malicious activities such as spoofing to proceed undetected. Model Stealing involves an attacker trying to clone the functionality of the target AI model [91,92]. In the context of ADS-B or RID, the attacker tries to fathom the inner workings of the AI model by sending a series of queries (malicious or benign ADS-B/RID messages) to the target system’s IDS and observing the corresponding output, which is typically the classification decision. This input/output behavior is then used to construct a surrogate model that functionally mimics the original IDS.

Additionally, model inversion attacks exploit the strong correlation between features and labels in highly predictive AI models to reconstruct sensitive features of the original training data [93,94,95]. In the context of ADS-B and RID, AI models are trained on massive datasets to learn patterns associated with legitimate operations and deviations, which a model inversion attack could infer those underlying, expected parameters, allowing attackers to craft ghost aircraft injection or spoofing attacks that closely mimic normal behavior, thereby making them harder to detect by anomaly detection systems. Membership Inference attacks aim to deduce whether a specific data record was used in the training dataset of the target ML model. It exploits the vulnerability of ML models to recall varying degrees of information about their training datasets. The attacker builds an attack model that is designed to distinguish between the output behaviors of the target model when presented with data it trained on versus data it did not train on. Finally, in the inference phase, the attacker queries the target model and uses the attack model to determine if a data record was in the training set [96,97,98]. This is used to craft an evasion attack to mimic those learned characteristics to avoid detection in ADS-B or RID IDS.

## 5. Attack Detection Techniques

Given that ADS-B and RID systems share comparable temporal characteristics, specifically the continuous, time-ordered broadcasts of flight state information, many threat detection frameworks developed initially for ADS-B can be effectively adapted to analyze RID data. While extensive research exists for ADS-B detection, these methodologies provide a critical baseline for securing emerging RID implementations. Hence, this section categorizes detection methodologies into non-AI approaches and AI-based strategies encompassing both traditional ML and DL, as shown in Table 7.

### 5.1. Non-AI Based Methods

Statistical and rule-based methods have been successfully deployed for anomaly detection, offering lower computational overhead, but they often lack the adaptability required to counter evolving, complex threats. Ref. [12] developed a multi-layered strategy combining flight plan validation, single-node detection (based on motion dynamics), and group-level detection to calculate an overall attack probability. Ref. [99] utilized a crowdsourced network to estimate Time Difference of Arrival (TDOA). By integrating this with a two-step Kalman filter, the system validates reported positions against kinematic parameters to detect GNSS tampering, spoofing, and false message injection. To detect replay attacks, ref. [100] proposed a data-driven method that computes cosine similarity between incoming ADS-B message sets and stored historical sets; sets whose similarity exceeds a specified threshold are flagged as potential replays. While these methods are cost-effective and do not require additional hardware, their reliance on predefined thresholds and expert rules limits their performance against sophisticated attacks [101,102].

### 5.2. AI-Based Methods

AI-based techniques offer superior adaptability, automatically learning complex spatial and temporal relationships to detect diverse anomalies.

#### 5.2.1. Traditional Machine Learning Methods

Traditional ML models, such as logistic regression, decision trees, k-nearest neighbors (KNN), Gaussian naïve Bayes, random forests, and support vector machines (SVMs), have been widely applied to ADS-B anomaly detection. Compared with rule-based or purely statistical approaches, these models tend to identify a broader range of attack behaviors and can offer improved detection efficiency for ADS-B and RID attacks.

In the jamming attacks, ref. [103] evaluated supervised learning methods (e.g., SVM and KNN) using features like Bit Error Rate and Signal-to-Noise Ratio. In addition, ref. [104] proposed a lightweight LightGBM approach optimized for UAV platforms, utilizing Spearman correlation for feature selection to reduce computational costs.

To detect message injection attacks, researchers have also applied a range of traditional ML techniques. For example, ref. [105] developed an SVM-based method to identify path modification, ghost aircraft injection, and velocity drift attacks simulated on the OpenSky network. Their approach involved data cleaning, encoding, standardization, and feature extraction, followed by experiments with Linear SVM, C-SVM, and Nu-SVM. Similarly, ref. [106] evaluated six supervised models for the same categories of ADS-B injection attacks, using selected operational features for training and testing. Moreover, ref. [107] proposed an ensemble meta-learning system that uses XGBoost (https://xgboost.readthedocs.io/en/stable/, accessed on 20 July 2025) and random forest as base learners, with logistic regression acting as the meta-classifier. To aid interpretation and support operational decision-making, the authors incorporated Local Interpretable Model-agnostic Explanations to provide local explanations of model predictions.

Beyond detecting individual attack categories, several studies have applied ML to identify multiple types of ADS-B attacks. For instance, ref. [108] proposed a dynamic temporal detection method based on a Sticky Hierarchical Dirichlet Process Hidden Markov Model. The sticky hierarchical Dirichlet process enables the model to infer parameters for the Hidden Markov Model dynamically, and the learned model predicts hidden states in ADS-B message sequences. The authors evaluated the approach on six attack types: constant deviation, random deviation, increased deviation, flight replacement, data replay, and denial-of-service. Ref. [109] utilized redundancy among geographically distributed sensors in a crowdsourced setting and introduced a hybrid scheme that combines verification checks with machine-learning classification of reception patterns. The system applies four verification tests (i.e., sanity, differential, dependency, and cross-checks) and then uses a Decision Tree classifier to determine message trustworthiness. In addition, ref. [33] extended ADS-Bsec by adding a machine learning module using SVM, decision trees, and random forests to classify replay attacks, ghost aircraft injection, and multiple ghost injection attacks. Ref. [69] constructed a dataset containing normal ADS-B traffic and four attack types (i.e., name jumping, false information, false heading, and false squawk) based on OpenSky data, following preprocessing steps such as missing-value handling, transformation, and outlier removal.

Although traditional ML models have shown strong performance in detecting specific categories of ADS-B attacks, their overall effectiveness remains limited. They often fail to capture the full range of abnormal behaviors present in real-world ADS-B traffic.

#### 5.2.2. Deep Learning Methods

DL models have recently shown strong effectiveness in capturing complex patterns and detecting anomalies in large-scale datasets. Their ability to automatically learn meaningful representations from raw or minimally processed data has led to increasing adoption in ADS-B security research. In this context, DL approaches have been applied to improve the detection of diverse attack types and to strengthen the overall security and resilience of both ADS-B and RID systems.

For the sequence modeling-based approach, ref. [110] used Long Short-Term Memory (LSTM) models to learn normal message sequences, identifying deviations as spoofing without requiring architectural changes to aircraft. Ref. [111] innovated by preprocessing messages into sliding windows of vectors, training an LSTM to predict future data and detecting residuals. Ref. [112] investigated various recurrent neural network architectures, including LSTM, Bidirectional LSTM, and GRU, specifically for detecting time-series injection attacks.

As for the other advanced architectures, ref. [113] proposed SODA, a two-stage deep neural network framework for detecting spoofing attacks in ADS-B systems. Ref. [114] proposed VAE-SVDD, an anomaly detection model that incorporates temporal correlations and distributional characteristics of ADS-B data. The method uses a variational autoencoder for data reconstruction and supports vector data description (SVDD) to learn the hypersphere boundary from reconstruction error. The authors evaluated the model using five simulated attack types, including constant and random position deviation, velocity drift, denial-of-service, and flight replacement. Ref. [115] proposed a novel approach by constructing an airspace flight image stream that integrates flight plan data with ADS-B information. They employed a generative adversarial network combined with LSTM to predict future flight images. Anomalies were detected by measuring the normalized cross-correlation between predicted and actual images, allowing for the identification and localization of abnormal targets. In addition, ref. [8] first provided a comprehensive review of existing machine learning and deep learning-based ADS-B intrusion detection methods, and then implemented three advanced DL models: TabNet, Neural Oblivious Decision Ensembles, and DeepGBM to classify ADS-B messages and identify specific attack types.

While many of the AI-based principles discussed for ADS-B are transferable to RID, the unique characteristics of RID’s broadcast protocols (e.g., Bluetooth and Wi-Fi) and its primary attack vectors (e.g., location spoofing and ghost injection) necessitate a distinct set of detection methodologies. Research in this area is nascent but critical.

Recent work has started to study RID-specific anomaly detection. Keizer et al. [116] proposed GhostBuster, which detects location falsification by comparing the position claimed in RID messages with an independent location estimate obtained from local sensing. An alarm is raised when the discrepancy exceeds a predefined bound. Sciancalepore et al. [117] presented ORION, which checks whether the sequence of RID-reported locations follows a set of allowed trajectories observed by distributed receivers. It triggers an alarm when the distance to the closest trajectory points exceeds a calibrated threshold. Beyond studies that target misbehaving UAVs, there is a limited number of publications related to on attacks on RID and their detection.

## 6. Countermeasures and Mitigations

Research on RID security countermeasures is currently fragmented compared to the mature, structured taxonomies available for ADS-B [26,27,28,118,119]. Unlike ADS-B, which has benefited from extensive taxonomy development and structured analysis over the last decade, RID efforts remain disparate [18,21,31]. Although ADS-B and RID use different protocols and operate in different environments, they face similar fundamental security challenges arising from their reliance on open wireless channels, largely unauthenticated broadcasts, and time-dependent state reports [20]. As a result, ADS-B research provides a useful reference for developing RID countermeasures. However, many techniques require RID-specific adaptation because RID uses Bluetooth/Wi-Fi broadcast stacks, involves heterogeneous receivers, and operates at shorter ranges. In this section, we review prior studies and relevant standards on ADS-B and RID, and for each countermeasure, we identify its security objective, key assumptions, and practical trade-offs (advantages and limitations). We organize the surveyed techniques by their primary mechanisms, including fingerprinting, anti-jamming, anti-spoofing, physical layer authentication (PLA) and physical layer security (PLS) methods, and AI-based mitigation, as illustrated in Table 8. These countermeasures offer complementary strengths, combining message-level integrity assessment with cross-sensor verification to enhance the robustness and trustworthiness of broadcast identification systems. Moreover, to provide a quantitative perspective, Table 9 summarizes the quantitative timing metrics that are reported in the surveyed ADS-B and RID security mechanisms.

### 6.1. Fingerprinting

Fingerprinting serves as a robust authentication technique by analyzing hardware imperfections or channel characteristics that are difficult for adversaries to replicate [21]. Fingerprinting methods are commonly categorized into three groups: channel/location-based, hardware-based, and software-based [124]. Hardware-based fingerprinting identifies transmitters by using physical characteristics such as radiometric features or clock skew. These methods work in controlled, short-range settings but are less reliable in dynamic, long-distance environments such as ADS-B and RID, and are vulnerable to attacks such as replay attacks. Software-based fingerprinting exploits vendor-specific implementations in device firmware, packet-generation timing, or protocol-handling behavior to identify legitimate nodes. Channel and location-based fingerprinting leverages signal strength and multipath profiles to cross-check reported locations against physical signal properties, effectively countering location spoofing in both ADS-B and RID [18,21,120,124]. Fingerprinting supports source authentication and attribution by linking ADS-B and RID transmissions to device-specific or channel-specific features, helping detect the identity and location of the spoofer.

For ADS-B, fingerprinting helps identify rogue transponders and verify that received messages originate from the claimed aircraft. For RID, it can differentiate legitimate UAVs from malicious impostors and counter spoofing attacks that attempt to broadcast fake identification or position messages. However, fingerprinting techniques often entail increased manufacturing costs and require specialized sensing devices, since their statistical nature demands a sufficient positive signal-to-noise ratio and stable measurements for reliable operation [37].

### 6.2. Anti-Jamming

Once interference or malicious signal manipulation is detected in ADS-B or RID broadcasts, mitigation options are relatively limited. Most existing countermeasures focus on suppressing or isolating the interfering signal so that the legitimate broadcast can still be received reliably. Physical-layer mitigation commonly relies on antenna-based techniques, such as forming spatial nulls with electronically steerable antenna arrays, or on signal-processing methods such as notch filtering. In antenna-based approaches, interference is reduced by steering a null toward the source of the malicious transmission while maintaining sufficient gain toward the legitimate aircraft or UAV transmitter. Prior work has explored a range of such solutions for GPS anti-jamming, varying in antenna technology, the signal attributes used for null formation, adaptation speed, and achieved suppression performance [125]. Other studies have proposed antenna designs intended to provide inherent resistance to jamming or spoofing, often assuming that suitable detection and adaptation algorithms can be integrated as needed [126,127,128,129]. Likewise, some work has focused primarily on adaptation algorithms without specifying the underlying antenna architecture. In parallel, several lightweight filtering and waveform-suppression methods have been examined. These processing-based countermeasures are typically effective only against particular interference types, yet they offer low computational and hardware complexity and may be feasible for deployment across a wide range of ADS-B and RID receivers [130,131,132]. These anti-jamming techniques primarily protect availability by mitigating interference so that ADS-B and RID broadcasts remain reliably receivable.

### 6.3. Anti-Spoofing

Many antenna-based mitigation strategies developed initially for jamming can also be applied to spoofing in ADS-B and RID systems. In these approaches, once the direction-of-arrival (DoA) of the interfering or spoofing signal is estimated, a spatial null is steered toward the hostile transmitter, thus suppressing the malicious signal while preserving the legitimate broadcast [99]. While techniques such as null-steering are possible, most signal-processing methods designed to reject jamming, such as narrowband notch filters, do not fully address wideband spoofing attacks that mimic legitimate transmissions. Consequently, spoofing mitigation often resorts to the detection of anomalous transmissions and the exclusion of the affected data, rather than active signal suppression [125]. Anti-spoofing methods aim to preserve message integrity by detecting or suppressing forged signals that mimic legitimate messages, often using direction or location consistency and statistical filtering.

### 6.4. Physical Layer Authentication (PLA) and Physical Layer Security (PLS)

For ADS-B and RID, physical layer defenses provide a complementary line of protection against spoofing and jamming by leveraging observable signal and channel characteristics [133]. In particular, PLA assesses whether a received broadcast is consistent with the claimed source using signal-derived or channel-derived features, offering a lightweight complement to higher-layer mechanisms for spoofing detection [121,134]. Secure location verification based on receiver-side DoA and ranging can help detect message injection and spoofing, and it can be realized by enhancing ADS-B In receivers without modifying the ADS-B protocol [50]. When channel conditions vary and attackers adapt, game-theoretic PLA provides a way to tune detection thresholds to balance false alarms and missed detections under spoofing [121]. In addition, physical layer security techniques such as beamforming, null steering, and artificial noise can improve resilience to interference and interception in UAV communications. For example, Zhou et al. [135] introduced a UAV friendly jammer that transmits artificial noise to improve the PLS of a legitimate transmitter-receiver pair if the eavesdropper location is unknown. Beyond bit-level confidentiality, semantic-layer PLS further shows that the semantic threshold controls the trade-off between reliable recovery of intended meaning at legitimate receivers and semantic leakage to eavesdroppers, which is relevant when an adversary targets identity and location semantics rather than exact payload bits [136]. PLA and PLS methods leverage signal and channel properties to verify the claimed transmitter or location, complementing cryptographic approaches without modifying legacy message formats.

### 6.5. AI-Based Mitigation

Given the demonstrated efficacy of AI in securing aviation communications, recent research has expanded beyond simple detection to explore active mitigation strategies for ADS-B and RID environments [122]. AI-based countermeasures enable the characterization of attack patterns, the inference of adversarial intent, and automated responses that reduce the operational impact of spoofing, jamming, or message manipulation.

ML is particularly effective for these systems because it can model the temporal broadcast patterns of ADS-B and RID data to identify deviations from expected behavior. Ref. [137] utilized DL to classify radio signals from large-scale real-world datasets, demonstrating that a Convolutional Neural Network (CNN) could effectively distinguish between ACARS and ADS-B transmissions. Similarly, ref. [122] proposed a machine-learning classifier capable of distinguishing various injection attacks to support automated mitigation strategies, such as adaptive navigation or strategic channel avoidance.

Ref. [123] introduced a distributed sensor framework for specific emitter identification (SEI) that extracts bispectrum-based fingerprint features and trains a CNN to distinguish individual ADS-B transmitters. To address the limited training data and annotation costs typical of SEI tasks, the authors apply a transfer-learning strategy that uses frozen parameters, maximum mean discrepancy, and classification-error constraints to reduce domain mismatch and improve robustness. A hyperbolic embedding module is added to strengthen feature representation. The resulting model generalizes well across heterogeneous sensor inputs, demonstrating how AI-based SEI can help mitigate spoofing and impersonation attacks in ADS-B systems.

In addition, ref. [138] emphasizes the importance of incorporating explainable AI (XAI) frameworks into UAV systems to make sure AI-driven decisions are presented in a transparent, human-understandable manner. Such capability allows operators to interpret, verify, and, when necessary, override autonomous behaviors, which is essential when responding to anomalies in UAVs. By combining XAI with decentralized learning mechanisms and emerging international AI safety standards, future UAV systems can achieve greater transparency and accountability, thereby strengthening trust and compliance in airspace environments that rely on ADS-B and RID for situational awareness and identification.

In the specific context of RID systems, AI and advanced cryptographic methods are being adapted to balance security with privacy. Ref. [7] introduced the Anonymous Remote Identification (ARID) protocol, a lightweight scheme using El-Gamal elliptic-curve cryptography and ECDSA. This allows for UAVs to broadcast messages that remain anonymous to the public while ensuring verifiability by trusted authorities. Ref. [26] extended this concept with the Anonymous Direct Authentication and Remote Identification ($A^{2}RID$) framework, proposing three variants ($CS-A^{2}RID$, $DS-CCA2-A^{2}RID$, and $DS-CPA-A^{2}RID$) tailored to varying UAV hardware and energy constraints. Other approaches leverage decentralized architectures for mitigation. Alkadi and Shoufan proposed a Mobile Crowdsensing model utilizing Ethereum smart contracts to enforce airspace regulations and verify drone identifiers, addressing the confidentiality and integrity limitations of current systems [27]. Finally, ref. [117] introduced ORION, a trajectory verification framework that compares real-time RID broadcasts against authorized waypoints, automatically raising alarms if deviations exceed offline-calibrated thresholds.

## 7. Existing Datasets and Tools

Effective research and development of ADS-B and RID security solutions rely on the availability of simulation tools, testbeds, and real-world or realistic datasets. In this section, we highlight key software tools, simulators, and data repositories that have been used in the community to analyze vulnerabilities, develop detection algorithms, and validate countermeasures. By compiling these resources, we aim to lower the barrier to entry for researchers in this domain and identify gaps where more data or better tools are needed.

### 7.1. Tools

Simulation tools offer a controlled and cost-effective virtual environment for cybersecurity research, enabling the investigation of vulnerabilities and the development of defense mechanisms without the risks associated with real-world flight testing. The selection of an appropriate simulator is based on research needs, functionalities, and applications of each tool. Table 10 summarizes various simulation tools relevant to ADS-B and RID. These simulation platforms can be broadly categorized into four principal types, these include network simulators, physical simulators, distributed co-simulators, and ground control station software [139].

Network simulators [140,141,142,143,144,145] are frameworks designed to model and analyze the communication protocols and network dynamics of UAV within a virtual setting which are particularly useful for investigating vulnerabilities in ADS-B and RID since they are primarily based on wireless communication. Prominent examples include NS3, OMNET++, GloMoSim, J-Sim, OPNET, NetSim, and QualNet. NS3 is particularly noted for its flexibility and modularity, although its lack of robust three-dimensional mobility models can limit its utility for UAV network research requiring spatial realism. OMNET++ is valued for its modular architecture and advanced visualization capabilities, making it suitable for a wide range of network simulation scenarios. QualNet stands out for its scalability and accuracy in simulating large-scale networks, which is especially advantageous for cybersecurity performance analysis.

Physical simulators [146,147,148,149] provide a visually realistic representation of the physical world by leveraging on high fidelity gaming engines. They can integrate advanced sensor models such as GPSs and LiDAR which are important for generating data feedback that approximates real-world conditions. This characteristic is useful in gathering environment-based training data for AI applications. Examples of physical simulators include Gazebo, Microsoft AirSim, MATLAB UAV Toolbox, and JMAVSim. Gazebo has been specifically enhanced to support UAV simulations; for instance, it was utilized in a methodology for generating datasets related to GPS spoofing attacks on UAV when combined with QGroundControl (QGC) and PX4 autopilot firmware [154].

Distributed Co-simulator [150,151,152,153] combines network and physical simulation capabilities, yielding a holistic platform for research. For instance, CUSCUS integrates NS-3 with a physical simulation module, facilitating both pure simulation and hardware-in-the-loop experimentation. Co-simulators are valuable to test interplay: e.g., how a drone reacts physically when its ADS-B or RID link is attacked and what the network does concurrently. They can also incorporate ground control station to simulate the operator’s perspective during these events.

GCS software are often deployed together with physical simulators to enhance operator control within the simulated environment. These include Mission planner, QGroundControl (QGC), Universal Ground Control Software (UGCS), and MAVPROXY. They can be used to observe how the GCS handles abnormal telemetry or to generate realistic command and control traffic for network analysis.

In summary, a rich set of tools exists, but one observation is that RID-specific simulation tools are still emerging. RID’s broadcast formats (i.e., Bluetooth 5, Wi-Fi NAN) are not natively supported in common network sims, so researchers might approximate them with custom models.

### 7.2. Datasets

ADS-B cybersecurity research relies on diverse data sources, each offering unique advantages for understanding system behavior, identifying vulnerabilities, and developing countermeasures. These datasets can be broadly categorized into Live or real-world ADS-B data and simulated normal and malicious data. Platforms like OpenSky Network [155], FlightAware, ADS-B Exchange, FlightRadar, FAA Database, ADS-BHub, and Aireon provide vast repositories of real-world ADS-B data, offering invaluable insights into global air traffic patterns. While real-world data is essential for understanding baseline behavior, it often lacks attack instances, which are crucial for training supervised ML models. Simulated and synthetic datasets bridge this gap by providing controlled environments where various attack scenarios can be generated and data labeled. For instance, the authors of [105] simulated several message injection attacks such as path modification, ghost aircraft injection, and velocity drift using real-world ADS-B messages from the OpenSky network. However, these simulations often present limited attack scenarios that are specific to the controlled environment in which they were generated, potentially failing to capture the full complexity and diversity of real-world cyber threats [156]. Additionally, these simulated data lack the complex physical layer impairments, such as multipath fading, hardware clock drifts, and dynamic channel noise, that characterize real electromagnetic environments. This creates a gap where ML models demonstrating high accuracy in controlled tests may suffer from poor generalization when exposed to the unpredictable signal characteristics of actual hardware based attacks. Table 11 provides summary of tools, databases and datasets relevant for ADS-B and RID research.

## 8. Challenges, and Future Research Directions

The integration of ADS-B and RID into modern aviation has undoubtedly improved operational awareness, but, as this survey has detailed, it also introduces significant cybersecurity challenges. In this final section, we discuss some of the overarching challenges and open research directions that remain. These include technical hurdles (e.g., securing broadcasts without overhauling legacy systems), operational and regulatory issues (e.g., deploying security enhancements and data sharing), and evolving threat paradigms (e.g., adversarial AI). Addressing these challenges is crucial for achieving a resilient air traffic and UAV traffic management ecosystem.

One of the fundamental challenges is that ADS-B is an established, widely deployed protocol (with a large installed base of transponders), while RID is being deployed in a cost-sensitive commercial drone market. Introducing robust security (like encryption or authentication) into these systems is non-trivial. Encryption and authentication techniques cannot be used in ADS-B and RID devices because their primary purpose is to broadcast aircraft position and status information so that it can be received and understood by any nearby aircraft and ground-based monitoring systems. ADS-B and RID are designed as an open, one-way broadcast system to enhance situational awareness, traffic separation, and overall aviation safety. If messages were encrypted or required authentication, only authorized receivers with the correct keys could decode them, preventing some aircraft or safety systems from accessing critical real-time information. This would undermine interoperability, delay information sharing, and reduce the effectiveness of collision avoidance and air traffic surveillance, which depend on immediate and universal data availability. Cryptographic solutions could solve many issues, but they face serious practical obstacles, including distributing and managing keys to every aircraft and drone and providing assurance that new secure messages can still be understood by legacy receivers. For ADS-B in particular, a complete switch to an encrypted protocol would likely mean retiring or upgrading tens of thousands of transponders, which is economically and logistically difficult. This leads to a stalemate in which we rely on external mitigations (such as those in Section 6) rather than fixing the protocol itself. Future research is exploring transitional methods, such as broadcasting an additional authenticated digest alongside ADS-B messages that could be ignored by old receivers but used by new ones for verification. However, even these introduce complexities, such as the need for a public key infrastructure and the risk of breaking the low-latency requirements of ADS-B. The aviation community is also concerned that adding security should not compromise the deterministic timing and low latency of ADS-B communications (which are critical for collision avoidance). Thus, one future direction is to develop lightweight authentication mechanisms tailored for ADS-B/RID constraints, possibly leveraging recent advances in efficient cryptography (e.g., short elliptic curve signatures) or even physical layer security techniques (e.g., embedding authentication in signal properties).

Another challenge is the escalating “cat-and-mouse” game of AI-driven security. As defenders deploy sophisticated AI-based methods for intrusion detection and anomaly detection, attackers are co-evolving. The new frontier is no longer simple spoofing but adversarial-aware attacks. Attackers are now leveraging formal AML techniques, such as evasion and poisoning attacks, to design attacks that are invisible to these new AI detectors. This co-evolution requires a change in research from static detection to robust, adversarially-trained, and dynamic defense models that can account for this attacker-in-the-loop threat.

Thirdly, the data desert for RID security, identified in this survey, creates a critical research bottleneck. As shown in Table 10, there is a near-total absence of large-scale, public, labeled datasets for RID-specific attacks targeting its unique Bluetooth and Wi-Fi broadcast mediums. This means that most, if not all, current AI-based detection research for RID is being performed on simulated, perfect-world datasets. These models are not being validated against the low-quality, intermittent, and short-range (e.g., 20 m) signals seen in practice. This disconnect means that current detection models are likely brittle and could fail when deployed. A fundamental challenge, therefore, is the creation of a new, realistic benchmark dataset for RID security.

Future research direction is to develop multi-layered and integrated security frameworks. Ultimately, addressing these challenges suggests that no single silver bullet will suffice. Instead, a multi-layered defense-in-depth approach is needed. For ADS-B and RID, this means combining preventive measures (e.g., future authentication protocols or signal encryption, if achievable) with detective measures (e.g., anomaly detectors and multilateration) and reactive measures, including procedures to follow when an attack is suspected (e.g., alerting air traffic control, or drones performing emergency landing if they detect compromise). It also means integrating both traditional techniques, such as radar, pilot vigilance, and modern AI-based techniques. The framework should also be scalable and interoperable, and should be able to cover dense urban drone traffic. Research needs to focus on how all these pieces can work together seamlessly. For instance, how to integrate an ADS-B spoofing alert from a ground system into the air traffic control decision chain quickly, or how a drone can securely fail-safe if its navigation is under attack without causing new hazards. Furthermore, it is essential to broaden the scope to include comprehensive cybersecurity assessments of RID systems. These evaluations should examine both protocol-level vulnerabilities and the wider ecosystem implications of potential attacks, especially given the growing use of UAV in delivery, infrastructure inspection, and emergency response activities.

## Figures and Tables

**Table 1:** ADS-B data fields, their purpose, and frequency of operations.

ADS-B Data Field	Purpose/Description	Periodicity
Preamble	Used to synchronize the transmitters and receivers	Static (≥1/1 s)
Downlink Format (DF)	Indicates the type of message. A value of 17 indicates the extended squitter message format	Static (≥1/1 s)
Transponder Capability (CA)	Indicates the communication capability of the Mode S transponder	Static (≥1/1 s)
ICAO Address (AA)	The unique 24-bit identifier assigned for the life of the transponder, used for aircraft identification	Static (≥1/1 s)
Message Field (ME)	Contains the corresponding surveillance data such as identification, position, velocity, urgency code, and quality level. The first bits are the type code (TC), which describes the type of information contained in the remaining 51 bits of the ME field.	Dynamic (≥1/1 s)
Parity Check (PI)	Used by receivers to detect and correct transmission errors	Dynamic (≥1/1 s)

**Table 2:** RID data fields, their purpose, and frequency of transmission.

RID Data Field	Purpose/Description	Periodicity
UAV ID	Unique identifier traceable to a unique UAV and its operator (Serial No., Reg. ID, UTM UUID, or Session ID).	Static (≥1/3 s)
Latitude/Longitude	Current geographical position of the UA.	Dynamic (≥1/1 s)
Timestamp	Time of applicability of the Location Message.	Dynamic (≥1/1 s)
Height Type	Height above takeoff location or above ground level (AGL).	Dynamic (≥1/1 s)
Operator Location	Location of the remote pilot/GCS, or the aircraft’s takeoff location if dynamic data is unavailable.	Static (≥1/3 s)
Velocity/Speed	Direction and speed of the UA.	Dynamic (≥1/1 s)
Emergency Flag	Operational status indicating distress or emergency.	Dynamic (≥1/1 s)
Horizontal/Vertical Accuracy	Quality/containment measure for positional data.	Dynamic (≥1/1 s)

**Table 3:** Summary of broadcast-based mediums, range covered, and characteristics.

Broadcast Medium	Range	Characteristics
Bluetooth Legacy (4.x)	∼400 m	Uses short “advertisement” frames, leading to limited range and requiring messages to be segmented.
Bluetooth 5.x Long Range	>1 km	Adds Forward Error Correction, increasing range by a factor of 4; requires use of the Message Pack format (Type 0xF).
Wi-Fi Neighbor Awareness Network (NAN)/Wi-Fi Aware	>2 km	Connectionless broadcast, enabling device exchange without infrastructure; payload encoded in Service Discovery Frame; power consumption ≈100mW.
Wi-Fi Beacon	>2 km	Encodes RID message pack as a vendor-specific information element payload; power consumption ≈100mW.

Note: Ranges cited from [22] represent rural/low interference environments. Urban ranges will be lower due to RF interference and obstruction.

**Table 4:** ADS-B and RID technical comparison.

Feature	ADS-B	RID
Data Link	Uses the Mode S Extended Squitter (1090ES) transponders and UAT.	Uses Bluetooth, Wi-Fi, or LoRa for BRID. Network RID uses LTE/5G for communication via dedicated servers.
Operating Frequencies	Mode S Extended Squitter: 1090 MHz. UAT: 978 MHz.	BRID uses unlicensed radio spectrum such as 2.4 GHz or 5.8 GHz. Net-RID uses the licensed radio spectrum allocated to the commercial cellular provider the UAV is subscribed to.
Message Broadcast Periodicity	Position data is broadcasted approximately once per second.	Dynamic messages shall be sent at least every second. Static messages shall be sent every three seconds.
Message Length	The Extended squitter messages have 112 bits with the embedded ADS-B data payload constituting 56 bits. UAT transmits dedicated ADS-B messages consisting of 272 bits.	Broadcast messages are designed to be short Block messages. Each broadcast message shall be 25 bytes in length: 1 byte header + 24 bytes data.
Key Message Content	Aircraft identification, position, altitude, velocity, and urgency code.	Unique identifier, UAV position, control station/remote pilot location, velocity, timestamp, and emergency flag.
Identity Broadcast	Has ICAO Address which is a fixed hardware ID and Call Sign as the flight ID.	Has a UAV ID which constitutes the UAV serial number and Session ID.
Range	High range, typically 185 km to 370 km.	BRID has a restricted local range of 200 m to 10 km. Net-RID coverage is limited to areas with network connectivity.

**Table 5:** Summary of existing ADS-B and RID survey papers.

Survey Paper	Working Principle		Attacks		Detection		Mitigations		Tools and Datasets	Research Gaps and Future Directions
	ADS-B	RID	ADS-B	RID	ADS-B	RID	ADS-B	RID		
[18]	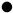	×	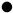	×	×	×	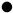	×	×	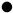
[12]	×	×	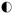	×	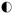	×	×	×	×	×
[30]	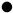	×	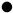	×	×	×	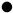	×	×	×
[31]	×	×	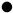	×	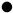	×	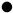	×	×	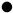
[32]	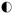	×	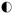	×	×	×	×	×	×	×
[33]	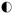	×	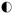	×	×	×	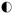	×	×	×
[11]	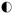	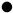	×	×	×	×	×	×	×	×
[20]	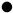	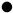	×	×	×	×	×	×	×	×
[14]	×	×	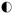	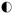	×	×	×	×	×	×
[34]	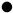	×	×	×	×	×	×	×	×	×
[35]	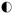	×	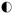	×	×	×	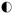	×	×	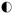
[15]	×	×	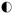	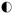	×	×	×	×	×	×
[36]	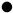	×	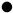	×	×	×	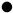	×	×	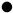
[37]	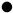	×	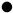	×	×	×	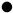	×	×	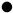
[38]	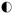	×	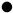	×	×	×	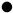	×	×	×
[39]	×	×	×	×	×	×	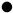	×	×	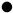
Our Paper	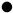	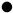	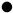	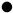	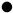	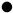	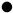	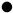	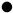	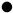

Legend: 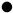 fully covered; 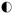 partially covered; × not covered.

**Table 6:** Summary of attacks, descriptions, and impacts on ADS-B and RID systems.

Attack Category	Attack Type	Description	Impact
Injection	ADS-B Injection	Injecting ADS-B messages into a data link, often by creating fake ADS-B signals or crafting messages that conform to the ADS-B protocol to mimic legitimate traffic.	Operational disruptions, Remote hijacking of aircraft, Increased risk of collisions and Mission failures.
	RID Injection	Injecting fake RID messages with the same unique identifiers.	Operational disruptions, Remote hijacking of aircraft, Increased risk of collisions and Mission failures.
	Ghost Injection	Injecting false aircraft or UAV data by broadcasting false ADS-B and RID messages, making it appear that real aircraft exists.	Increased risk of collisions, Inaccurate data collection, and data integrity compromise.
DoS/DDoS	Jamming	Signals are transmitted by the jammers to prevent the reception of ADS-B/RID messages or GPS signals.	Communication loss, loss of UAV control, mission failure, collision risk.
Intrusion	Malware Infection	Malicious software is covertly installed on ADS-B/RID module’s processing unit.	Communication loss, loss of UAV control, mission failure, collision risk, data corruption.
	Firmware flashing/modification	Unauthorized alteration or replacement of embedded firmware residing in ADS-B and RID modules to modifications that bypass integrity checks, disable logging or even forge legitimate broadcasts.	Operational, disruptions, hardware/software malfunction, mission failure, collision.
	Fuzzing	Intentionally providing malformed or random inputs targeting communication channels of ADS-B and RID devices with the goal of discovering protocol vulnerabilities in how ground control stations, Command and Control systems or traffic management software parse and process incoming messages.	Operational disruptions, collision risk, mission failure.
Spoofing	GPS Spoofing	Broadcasting fabricated GPS signals to cause the target GPS receiver to calculate an incorrect position, which is then broadcast via ADS-B or RID devices.	Incorrect navigational data, mission failure, risk of collision.
	RID Spoofing	Broadcast of falsified identification packets intended to impersonate legitimate UAVs to mislead both observers and neighboring UAVs.	Inaccurate broadcast data, compromised UAV identification, risk of collision, mission failure.
	ADS-B Spoofing	Broadcasting counterfeit ADS-B signals to impersonate legitimate aircraft and induce incorrect positioning or timing information.	Inaccurate broadcast data, compromised aircraft situational awareness, risk of collision, mission failure.
AitM	Replay	Capturing legitimate messages (ADS-B or RID) and re-transmitting them later with the intent to deceive.	Operational disruptions, data corruption, Collision risk, mission failure.
	MitM	Intercept, modify, change or even replace messages of either ADS-B or RID, thereby compromising the integrity of their messages.	Data corruption, hijack, collision risk, mission failure.
Supply Chain	Hardware Supply Chain	Malicious modifications are introduced to hardware components of ADS-B or RID modules at any stage of the supply chain; from design to manufacturing and distribution.	Persistent unauthorized access and control of hardware components, prolonged operational disruptions, mission failure, collision risk.
	Software Supply Chain	Compromising a software that processes ADS-B/RID data by injecting malicious code into the software at any point in its lifecycle, such as during design, development, deployment, or updates.	Persistent backdoor to software, prolonged operational disruptions, mission failure, collision risk.
AML	Poisoning	Corrupts datasets used for training models for anomaly detection in ADS-B or RID or introduces corrupted sensor data.	Data corruption, path deviation, collision risk, mission failure.
	Evasion	Deceives AI models for anomaly detection into making incorrect classifications by subtly manipulating the input test data.	Data corruption, path deviation, operational disruptions, collision risk, mission failure.
	Model Stealing	Duplicates or clones the functionality of the target AI model using input/output behavior to construct a surrogate model that functionally mimics the AI-based detection systems.	Data corruption, path deviation, operational disruptions, collision risk, mission failure.
	Model Inversion	Reconstructs sensitive features of training data by exploiting the correlation those features have with the model’s output.	Data corruption, Path deviation, Operational disruptions, Collision, Mission Failure.
	Membership Inference	Attempts to infer whether a specific data record was included in the training dataset of a target AI model in order to craft evasion attack to mimic those learned characteristics to avoid detection.	Data corruption, path deviation, operational disruptions, collision risk, mission failure.

**Table 7:** Comparison of attack detection techniques for ADS-B and RID.

Category	Typical Targeted Attack Types	Strengths	Limitations
Non-AI	GNSS spoofing and tampering, false position injection, data replay, ghost aircraft, coarse trajectory anomalies.	Low computational overhead; simple to implement; no need for labeled datasets; can be integrated with existing ADS-B/RID infrastructure.	Depend on fixed thresholds and expert rules; limited adaptability to complex or evolving attacks; reduced effectiveness in dense or atypical traffic.
AI-based (traditional ML)	Message injection and modification, path changes, ghost aircraft injection, velocity drift, denial-of-service, and jamming detection.	Detect a broader range of attack behaviors than rule-based methods; improved detection efficiency reported in several studies; lightweight variants exist for UAV platforms with feature selection.	Require labeled or carefully prepared datasets and feature engineering; performance may degrade when traffic patterns or attack behavior change.
AI-based (DL)	Position and velocity deviation, velocity drift, DoS, flight replacement, and multiple simulated ADS-B attack types.	Learn temporal and distributional characteristics directly from data; effective on diverse attack scenarios in reported simulations.	Require more training data and parameter tuning than non-AI methods; typically higher computational complexity.

**Table 8:** Comparison of countermeasure categories for ADS-B and RID.

Category	Typical Objectives/ Attack Types	Strengths	Limitations
Fingerprinting	Authenticate transmitters and distinguish legitimate ADS-B/RID emitters from rogue or spoofed ones; counter identity and location spoofing.	Uses hardware, software, or channel/location features that are difficult to replicate; helps identify rogue transponders and verify that messages originate from the claimed aircraft or UAV.	Often reliable only in short-range or controlled environments; require specialized sensing devices and sufficient SNR; increase manufacturing and hardware cost.
Anti-jamming	Mitigate interference and intentional jamming that degrade ADS-B/ RID broadcasts.	Antenna arrays and signal-processing methods (e.g., spatial null steering, notch filtering, waveform suppression) can attenuate interference while preserving the legitimate broadcast; lightweight filtering methods offer low computational and hardware complexity.	Typically tuned to specific interference types; less effective for wideband or sophisticated jamming; some techniques assume advanced antenna architectures not available on all receivers.
Anti-spoofing	Suppress or filter spoofed ADS-B/RID transmissions that mimic legitimate broadcasts.	DoA–based null steering can place a spatial null on the spoofing source while preserving desired traffic; many anti-jamming antenna strategies can be reused for spoofing scenarios.	Narrowband filtering does not fully address wideband spoofing that closely imitates legitimate signals; in practice, mitigation often relies on detecting anomalous transmissions and discarding affected data rather than fully cancelling the spoofed signal.
PLA & PLS	Mitigate spoofing, message injection, and jamming.	Leverages channel, signal, and location features for authentication; enables receiver-side DoA or ranging checks without ADS-B protocol changes; PLS techniques (e.g., beamforming, artificial noise, and null steering) mitigate interference and leakage.	Depends on channel conditions and receiver capability; limited in long-range/low-cost settings; some PLS assumes directional/control links and is less suited to open broadcast.
AI-based mitigation	Support mitigation for spoofing, jamming, message injection, and impersonation; strengthen RID trust and privacy.	ML/DL models can characterize attack patterns, infer adversarial intent, and trigger actions such as adaptive navigation or channel avoidance; AI-based SEI improves identification of individual transmitters; anonymous RID protocols and decentralized enforcement enhance verifiability and compliance while protecting operator privacy.	Require training data, model tuning, and additional processing compared with basic methods; cryptographic and decentralized schemes introduce key-management and communication overhead and must be tailored to UAV hardware and energy constraints.

**Table 9:** Quantitative timing results reported in the literature for ADS-B and RID security mechanisms.

Reference	System	Reported Timing Metric
[120]	ADS-B	≈6–9 s prediction time.
[121]	ADS-B	GNSS jamming detection time < 15 s.
[122]	ADS-B	Not reported.
[123]	ADS-B	Not reported.
[7]	RID	ARID takes ≈11.23 ms to generate a message.
[26]	RID	$CS-A^{2}RID\approx 17.34$ ms to generate anonymous RID messages; $DS-CCA2-A^{2}RID\approx 12.05$ ms to generate anonymous RID messages; $DS-CPA-A^{2}RID\approx 5.95$ ms to generate anonymous RID messages.

**Table 10:** Summary of tools useful for ADS-B and RID cybersecurity research.

Category	Simulators	Description
Network Simulators [140,141,142,143,144,145]	NS3 (https://www.nsnam.org/, accessed on 20 June 2025); OMNet++ (https://omnetpp.org/, accessed on 20 June 2025); GloMoSim (https://github.com/ykzeng/glomosim/tree/master, accessed on 20 June 2025); JSim (https://github.com/martimy/JSim, accessed on 20 June 2025); OPNET (https://opnetprojects.com/opnet-network-simulator/, accessed on 20 June 2025); NetSim (https://netsim.boson.com/, accessed on 20 June 2025); QualNet (https://www.keysight.com/us/en/assets/3122-1395/technical-overviews/QualNet-Network-Simulator.pdf, accessed on 20 June 2025).	Tools for analyzing network behavior and communication protocols.
Physical Simulators [146,147,148,149]	AirSim (https://airsim-fork.readthedocs.io/en/docs/, accessed 20 June 2025); MATLAB UAV Toolbox (https://www.mathworks.com/products/uav.html, accessed on 20 June 2025); Gazebo (https://gazebosim.org/home, accessed on 20 June 2025); JMAVSim (https://github.com/PX4/jMAVSim, accessed on 20 June 2025); PX4 SITL (https://docs.px4.io/main/en/simulation/, accessed on 20 June 2025); FlightGear (https://www.flightgear.org/, accessed on 20 June 2025); X-Plane (https://www.x-plane.com/, accessed on 20 June 2025).	Platforms for simulating UAV flight dynamics, sensor data, and environmental interactions.
Distributed Co-simulators [150,151,152,153]	CUSCUS (https://www.sciencedirect.com/science/article/abs/pii/S1570870517301701, accessed on 20 June 2025); AVENS (https://www.lsec.icmc.usp.br/en/avens, accessed on 20 June 2025); ROS-NetSim (https://github.com/alelab-upenn/ros-net-sim, accessed on 20 June 2025); FlyNetSim (https://github.com/saburhb/FlyNetSim, accessed on 20 June 2025); RoboNetSim (https://www.sciencedirect.com/science/article/pii/S0921889013000080, accessed on 20 June 2025); CORNET (https://ieeexplore.ieee.org/document/9027459, accessed on 20 June 2025); SUMO (https://sumo.dlr.de/docs/index.html, accessed on 20 June 2025).	Combines network and physical simulations.
Ground Control Station Software	Mission Planner (https://ardupilot.org/planner/, accessed on 20 June 2025); QGC (https://qgroundcontrol.com/, accessed on 20 June 2025); UGCS (https://www.sphengineering.com/flight-planning/ugcs, accessed on 20 June 2025); MAVProxy (https://ardupilot.org/mavproxy/index.html, accessed on 20 June 2025).	Software for planning, monitoring, and controlling UAV missions.

**Table 11:** Publicly available ADS-B and RID databases.

Name/Reference	Target	Year	Data Role	Attack Type	Number of Features	Number of Records	Nature
OpenSky [157]	ADS-B	Continuous	Baseline (Normal Traffic)	ADS-B Message Injection/ Modification	17	Variable	Real-time
FlightAware [158]	ADS-B	Continuous	Baseline (Normal Traffic)	ADS-B Message Injection/ Modification	20	Variable	Real-time, Historical
ADS-B Exchage [159]	ADS-B	Continuous	Baseline (Normal Traffic)	ADS-B Message Injection/ Modification	To be extracted based on need	Variable	Real-time
FlightRadar24 [160]	ADS-B	Continuous	Baseline (Normal Traffic)	ADS-B Message Injection/ Modification	To be extracted based on need	Variable	Real-time
ADS-BHub [161]	ADS-B	Continuous	Baseline (Normal Traffic)	ADS-B Message Injection/ Modification	To be extracted based on need	Variable	Real-time
Aireon [162]	ADS-B	Continuous	-	ADS-B Message Injection/Modification	To be extracted based on need	-	Real-time
UAV Dataset under Normal and Cyber-Attacks [163]	RID	2023	Pre-labeled Attack/Legitimate	Wi-Fi Deauth DoS, Replay, FDI, Evil Twin	Physical Dataset: 16, Cyber Dataset: 37	-	Live (Physical Tesbed)
ECU-IoFT [164]	RID	2022	Pre-labeled Attack/Legitimate	Wi-Fi Deauth, WPA2-PSK Cracking	-	-	Live (Physical Tesbed)
DeepSim [97]	GPS	2020	Pre-labeled Attack/Legitimate	GPS Spoofing	Aerial photos, satellite imagery	967	Live (Physical Tesbed)
UAV Attack Dataset [165]	GPS	2020	Pre-labeled Attack/Legitimate	GPS Spoofing, GPS Jamming, Ping DoS	Physical Dataset: 16, Cyber Dataset: 37	Cyber 53,976, Physical 54,784	Simulated and live flight data
Dataset for GPS Spoofing [166]	GPS	2022	Pre-labeled Attack/Legitimate	GPS Spoofing	13	158,170	Simulated
GPS Spoofing Detection Dataset [167]	GPS	2023	Pre-labeled Attack/Legitimate	GPS Spoofing	25	37,506	Simulated
[168]	GPS	2024	Pre-labeled Attack/Legitimate	GPS Spoofing	44	-	Live (Physical Tesstbed)

## Data Availability

No new data were created or analyzed in this study.
